# Particulate Matter and Gaseous Pollutions in Three Metropolises along the Chinese Yangtze River: Situation and Implications

**DOI:** 10.3390/ijerph15061102

**Published:** 2018-05-28

**Authors:** Mao Mao, Xiaolin Zhang, Yan Yin

**Affiliations:** Key Laboratory of Meteorological Disaster of Ministry of Education, Joint International Research Laboratory of Climate and Environment Change, Collaborative Innovation Center on Forecast and Evaluation of Meteorological Disasters, Key Laboratory for Aerosol-Cloud-Precipitation of China Meteorological Administration, Nanjing University of Information Science & Technology, Nanjing 210044, China; mmao@nuist.edu.cn (M.M.); yinyan@nuist.edu.cn (Y.Y.)

**Keywords:** fine particulate matter, trace gases, Yangtze River, metropolises

## Abstract

The situation of criteria atmospheric pollutants, including particulate matter and trace gases (SO_2_, NO_2_, CO and O_3_), over three metropolises (Chongqing, Wuhan, and Nanjing), representing the upstream, midstream and downstream portions of the Yangtze River Basin from September 2015 to August 2016 were analyzed. The maximum annual mean PM_2.5_ and PM_10_ concentrations were 61.3 and 102.7 μg/m^3^ in Wuhan, while highest annual average gaseous pollutions occurred in Nanjing, with 49.6 and 22.9 ppb for 8 h O_3_ and NO_2_, respectively. Compared to a few years ago, SO_2_ and CO mass concentrations have dropped to well below the qualification standards, and the O_3_ and NO_2_ concentrations basically meet the requirements though occasionally is still high. In contrary, about 13%, 25%, 22% for PM_2.5_, and 4%, 17%, 15% for PM_10_ exceed the Chinese Ambient Air Quality Standard (CAAQS) Grade II. Particulate matter, especially PM_2.5_, is the most frequent major pollutant to poor air quality with 73%, 64% and 88% accounting for substandard days. Mean PM_2.5_ concentrations on PM_2.5_ episode days are 2–3 times greater than non-episode days. On the basis of calculation of PM_2.5_/PM_10_ and PM_2.5_/CO ratios, the enhanced particulate matter pollution on episode days is closely related to secondary aerosol production. Except for O_3_, the remaining five pollutants exhibit analogous seasonal patterns, with the highest magnitude in winter and lowest in summer. The results of back trajectories show that air pollution displays synergistic effects on local emissions and long range transport. O_3_ commonly demonstrated negative correlations with other pollutants, especially during winter, while moderate to strong positive correlation between particulate matter and NO_2_, SO_2_, CO were seen. Compared to pollutant substandard ratios over three megacities in eastern China (Beijing, Shanghai, and Guangzhou), the situation in our studied second-tier cities are also severe. The results in this paper provide basic knowledge for pollution status of three cities along Chinese Yangtze River and are conductive to mitigating future negative air quality levels.

## 1. Introduction

Along with rapid industrial development and urbanization, China has witnessed a fast growing economy as well as increasingly persistent serious haze-smog during the past three decades. Outdoor atmospheric pollution is a mixture of particulate matter and trace gases, and sustained long-term exposure to high air pollution level has been extensively associated with a series of health hazards to the Chinese population, including acute mortality, morbidity, respiratory, and cardiovascular symptoms and damages, especially in densely populated cities [1,2,3,4]. On the basis of the Global Burden of Disease Study, air pollution has been considered as the high priority factor, responsible for 1.37 million premature Chinese deaths in 2013 [5]. Rohde et al. pointed out that about 1.6 million particulate matter-related premature death cases (17% of all reported deaths) occurred in 2014 [6]. Song et al. estimated that 1.52 million adults died prematurely related with outdoor particulate matter pollution over China in 2015 [4], and it ranked fourth in leading risk factors after heart disease, dietary risk and smoking [7]. Complex air pollution characterized by the strengthening of both atmospheric oxidation capacity and secondary aerosols has become more common in China [8]. Researchers have made a striking effort to investigate sources, characteristics, mechanism and adverse health effects of air pollution in China during the last 10 years, chiefly concentrating in three major city clusters: the Beijing-Tianjin-Hebei region, the Pearl River Delta and the Yangtze River Delta (YRD).

Since 1 January 2013, the Ministry of Environmental Protection (MEP) of China started to release official revisions of the ambient air quality index (AQI) at each air-monitoring stations in some major cities (163) based on the level of ground six criteria pollutants, namely particulate matter with aerodynamic diameter ≤ 2.5 μm (PM_2.5_), particulate matter with aerodynamic diameter ≤ 10 μm (PM_10_), ozone (O_3_), nitrogen dioxide (NO_2_), carbon monoxide (CO), and sulfur dioxide (SO_2_). By the end of 2014, the government has established more than 1436 national air quality monitoring sites in 367 cities for real-time and publicly accessible air quality observation data on above six pollutants. Thus, we could improve our understanding of issues like emission sources, formation mechanisms, transport pathway, and possible reasons for the remarkable decline in air quality by using this extensive dataset. Compared with most previous researches, the real-time release of ground-based monitoring data on the web-platform provides a powerful data source with more monitoring sites for a city, therefore, better reflecting the atmospheric pollution concentrations and corresponding urban air quality condition. Additionally, the data also supply continuous monitoring of air pollutants, enabling us to explore the seasonal and inter-annual dynamic changes between and within cities.

The Changjiang (also named the Yangtze River) is the longest river in Asia and the third-longest in the world, with a length of 6380 km, flowing entirely within China. It drains one-fifth of the land area and its river basin is home to nearly one-thirds of the Chinese population. For thousands of years, the river has been used for water, irrigation, sanitation, transportation, industry, and boundary-marking. Flanked by metallurgical, power, chemical, auto, building materials and machinery industrial belts and high-tech development zones, the Yangtze River is playing an increasingly crucial role in the economic growth of its basin and has become a vital link for international shipping to the inland provinces. In mid-2014, the Central Government of China launched the program called the Yangtze River Economic Belt, covering about 2 million square kilometers alongside the river, which has accelerated development of the Yangtze River urban agglomerations, and is being carried out in full swing. Inevitably, with increasing of population and economic development, the air quality in Yangtze River Basin is not optimistic. For example, according to the statistic of the meteorological department in Nanjing, from 1961 to 2013, the annual occurrence of haze days in Nanjing has been increasing at a rate of 42.9 days/decade [9]. The occurrence of haze days was 242 days, and winter average concentrations of PM_2.5_ were observed over 158.5 μg/m^3^ with significant degradation of visibility in 2013 [10], posing a potential influence on local meteorology and air quality.

Chongqing, Wuhan and Nanjing, located in the upper, middle and lower reaches of the Yangtze River, respectively, were chosen for this study as the most representative fast growing metropolises to evaluate air pollution situation in the Yangtze River Basin. These second-tier cities are hot spots of studied regions due to rapid economic development, urbanization of high population density, and relatively high emission densities of air pollutants, such as PM_2.5_, black carbon, and polycyclic aromatic hydrocarbons, which indicate severe particulate matters pollution and the detrimental health impacts. The question to be answered by this study was what is the pollution situation in these second-tier cities over the Yangtze River Basin, lighter or more serious, compared with the megacities in China, i.e., Beijing, Shanghai, and Guangzhou. Surveys of long-term trends in the spatiotemporal variations of six criteria pollutant concentrations over the whole Yangtze River Basin, are still scarce in the literature. To fill this knowledge gap, we present our concurrent studies of particulate matter and gaseous pollution levels in three typical cities from 2015 to 2016. The obtained corresponding knowledge of the three representative metropolises in this study should be very useful for policy makers to insightfully investigate the air pollution situation, and design effective pollution control strategies for the regional air quality management on the future development of other hot-spot cities in the Yangtze River Basin.

## 2. Methods

### 2.1. City Description

**Figure 1:**
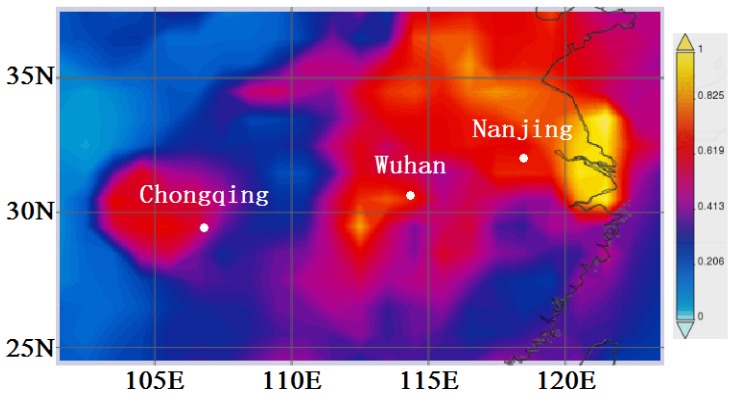
Aerosol optical depth (AOD) map of area-averaged time series over the Yangtze River Basin in China derived from MODIS-Aqua data from 1 September 2015 to 31 August 2016. Three white solid circles denote the cities of Chongqing, Wuhan, and Nanjing, respectively.

The geographical locations of the three cities are shown in Figure 1, and other information such as altitude, area, death rate, gross domestic product (GDP), population, vehicle counts are listed in Table 1. Meteorological conditions in the three cities are similar, and they belong to the subtropical climate zone with a distinct four season pattern, i.e., early spring, hot summer, cool autumn, and a cold winter.

Chongqing (center: 106.9’ E, 29.4’ N), one of the four provincial-level municipalities directly under the central government, has a long history and serves as the economic centre of the upstream region of the Yangtze River. Meanwhile, it is also a representative city in the Sichuan Basin of southwestern China. The Yangtze River runs through its whole area from west to east, covering a course of 665 km. The whole area slopes down from north and south towards the Yangtze River valley, with sharp rises and falls. As a result of the mountainous topography (north: the Daba Mountains, east: the Wu Mountains, southeast: the Wuling Mountains, south: the Dalou Mountains), air circulation in this typical valley city is blocked and the climate is wet and fogy. Chongqing is within the region of lowest wind speed over China (annual averages of 0.8–1.6 m∙s^–1^) (http://www.cqtj.gov.cn), sometimes being affected by calm winds, which favors the accumulation of pollutants. There is a total population of 30.5 million in its metropolitan area in 2016, which is the most among the provincial capital cities in China. It often rains at night in late spring and early summer, and thus the city is famous for its ‘night rain in the Ba Mountains’. With heavy industry as the mainstay industry, Chongqing is one of the oldest industrial bases in China and has a large demand on energy sources, more than 70% of which is coal [11].

Wuhan (center: 114.3’ E, 30.6’ N), the capital city of Hubei Province, is a representative large city situated on the eastern part of the Jianghan Plain in the midstream region of the Yangtze River, where the Han River flows into the Yangtze River and is surrounded by mountains on three sides. It covers an area of around 8494 km^2^ with a total number of residents of approximately 10.8 million in 2016. Wuhan is an important industrial base in China, possessing a complete and flourishing industrial system in the urban area, such as steel, automobile, photoelectron, chemical, metallurgy, textile, shipbuilding, manufacturing and medicine. Wuhan, with a GDP of 1191.3 billion Chinese currency, ranking number 1 in Hubei Province and number 9 in China in 2016, is undergoing rapid urbanization and economical development. Associated with areas of water bodies within its territory totaling 25.8%, Wuhan currently suffers from severe atmospheric problems from highway and river transportation, and the presence of different industrial activities along the Yangtze River.

Nanjing (center: 118.8’ E, 32.1’ N), the capital of Jiangsu Province, with an area of 6587 km^2^ and more than 8.27 million inhabitants at the end of 2016, is located in the downstream reaches of the Yangtze River. Being the second largest commercial center in the YRD region after Shanghai, Nanjing is highly urbanized and industrialized. Frequent occurrence of haze days and severe aerosol pollutions were observed from historical data. Considering the high density of population, adverse impacts on human health can be expected [12,13]. Nanjing is facing considerable pressure to restore its natural environment and environmental protection from anthropogenic destruction.

### 2.2. Date Source

One-year data spanning from 1 September 2015 to 31 August 2016 were analyzed in this study. All the measurements were conducted at the National Environmental Monitoring (NEM) sites for each city, and automated monitoring systems were constructed to measure 6 criteria pollutants according to China Environmental Protection Standards. The corresponding hourly mass concentrations of six pollutants in each monitoring site were reported to the NEM Center of China, and then released on the official website of the Chinese Environmental Protection Bureau (http://www.cnemc.cn) after verification in accordance with the Technical Guideline on Environmental Monitoring Quality Management.

Designed as a mix of urban sites (majority) and background sites (a few) for the point layout of ambient air quality monitoring, presently there are 17, 10, and 9 monitoring sites in Chongqing, Wuhan, and Nanjing, respectively (Table 1). The environmental conditions near the sampling sites, e.g., emission sources and underlying surface characteristics, are relatively stable. Mass concentrations of PM_2.5_ and PM_10_ were measured using the micro-oscillating balance and the β absorption method. Trace gases, including NO_2_, SO_2_, CO and O_3_, were measured using a chemiluminescence method, the ultraviolet fluorescence method, the filter infrared absorption method (or the non-dispersion infrared absorption method), and the ultraviolet spectrophotometry method, respectively. We note that in this work the gas species were converted from reported units to ppb or ppm using the ideal gas law and assuming a constant pressure at 1 atm. The quality assurance and controls of the state-controlled monitoring stations were reported in previous studies [14,15].

The meteorological observation data, including air temperature, pressure, relative humidity (RH), wind speed, visibility and precipitation, were obtained from the Chinese meteorological datum station. All the meteorological parameters have eight measurements per day, conducted at 02, 05, 08, 11, 14, 17, 20, and 23, local hour. Daily average meteorological parameters were then scalar averaged (excepted precipitation) and accumulated (precipitation) using the eight measurement results.

### 2.3. Backward Trajectory

To track the general transport characteristics of airborne masses recorded at the sampling sites, the backward trajectories were calculated and clustered using the Hybrid Single-Particle Lagrangian Integrated Trajectory Model (HYSPLIT), developed in the Air Resources Laboratory (ARL) of the USA National Oceanic and Atmospheric Administration (NOAA) [16]. Trajectories extending 72 h into the past were calculated four times every day (0, 6, 12, and 18 UTC) to examine the histories of air masses for the study period. Each trajectory was estimated at 500 m above ground level. Seasonal trajectories were classified using the hierarchical clustering method [17].

## 3. Results and Discussion

### 3.1. Overview of Air Pollution

The regional distribution of yearly mean aerosol optical depth (AOD) was derived from Giovanni maps with the Moderate Resolution Imaging Spectroradiometer (MODIS) satellite data [18]. Figure 1 illustrates intense values of AOD in the Yangtze River Basin in China, especially over the Sichuan Basin, Jianghan Plain, and Yangtze River Delta (YRD). The higher AOD values indicate higher aerosol loading, and are related to severe air pollution effects such as regional brown hazes largely contributed by anthropogenic emissions or dust storms caused by natural sources [19,20,21]. Mean AOD at 550 nm during the whole studied period was 0.58, 0.68, and 0.62 over Chongqing, Wuhan, and Nanjing, respectively.

The concentrations at all sites in every city are averaged to obtain the citywide average concentrations. The statistics of PM_2.5_, PM_10_, 8 h peak O_3_ (8 h-averaged O_3_), NO_2_, CO and SO_2_ averaged over the entire study time period for Chongqing, Wuhan, Nanjing are listed in Table 2. Particulate matter is a kind of environmental pollution closely related to the human health and the climate of the Earth. PM_2.5_ is known as fine particles, and particles with an aerodynamic diameter between 2.5 and 10 micrometers (PM_2.5–10_) is called coarse particle. The annual mean PM_2.5_ concentrations in Chongqing, Wuhan and Nanjing are 50.3 ± 25.3, 61.3 ± 40.9, and 53.7 ± 35.6 μg/m^3^, respectively, while the annual mean PM_10_ concentrations in Chongqing, Wuhan and Nanjing are 73.9 ± 32.0, 102.7 ± 53.3, and 94.0 ± 51.4 μg/m^3^, respectively. In comparison with the revised second grade of Chinese Ambient Air Quality Standards (CAAQS-II, GB3096-2012) for annual mean (PM_2.5_: 35 μg/m^3^; PM_10_: 70 μg/m^3^) [22] and the guideline recommended by World Health Organization (PM_2.5_: 10 μg/m^3^; PM_10_: 20 μg/m^3^) [23], both PM_2.5_ and PM_10_ levels have severely exceeded the limit, highlighting a high health risk. Based on the annual average values, Wuhan and Nanjing in the middle and lower reaches of the Yangtze River have worse air quality than Chongqing in upper Yangtze River. This may be attributable to some specific feature of local/regional emission sources mixed with meteorology influence.

Unprecedented levels of economic growth, the development of large-scale industries and services, and the boom of the vehicular population that has taken place in these regions have rendered air pollution an important and timely issue. Firstly, one of the mainly sources of particle pollution is coal. China is the largest coal consuming country in the world, with an absolute proportion of 50%. The survey results showed that coal burning by power plants, industrial processes, and domestic heating/cooking combined accounted for 40% of the population-weighted ambient PM_2.5_ concentration and was responsible for 60% of the air pollution-related health impacts in China [24]. Anthropogenic emissions have to a certain extent been controlled well with the arrival of ‘Action Plan for the Control of Air Pollution’ document issued by the Chinese government in September 2013. Subsequently, MEP announced the ‘Action Plan for Clean and Efficient Utilization of coal 2015–2020’ to reduce coal consumption in year 2015. Rural Chongqing and nearby Guizhou Province were reported to be typical areas of endemic arsenic pollution related to local high-arsenic coal production and combustion [25]. Chen et al. [26] analyzed that coal combustion represented a mean contribution of 22% to PM_2.5_, which was the largest of all the identified primary sources in Chongqing by employing Positive Matrix Factorization model when Chongqing burnt 60.96 million tons of coal in 2014, about 1.5% of the national coal consumption. With the specific measures for total coal consumption control, in 2015, the national total energy consumption was 4.3 billion tons of standard coal, up by 0.9% compared with the previous year, but the percentage of coal consumption was 64%, down by 3.7% against 2014, and the percentage of clean energy consumption was 17.9%, up 1% compared to 2014. The Yangtze River Basin was quite effective in eliminating coal-fired boilers and furnaces or retrofitting them to using clean energy. For instance, by the end of 2015, Chongqing had accomplished its goal of 275,000 kWh for eliminating outdated small coal-fired units. In Jiangsu Province, the electric-power industry eliminated outdated production capacity by 526,250 kWh, achieving most of its goal (527,000 kWh), and meanwhile, Jiangsu Province eliminated most of the small coal-fired boiler within the central heating zone, accomplishing the target for 2015 of eliminated 3100 small coal-fired boilers [24]. In recent years, some heavy industries, e.g., iron, steel and cement with the highest density of coal consumption, have been gradually moved away from urban areas to the suburbs to reduce emissions, especially in the valley-based Chongqing region [15].

Secondly, automobiles that mostly fall short of emission standards, and dust from roadwork and urban construction projects are another two kinds of air pollution sources. Maximum concentrations are reached during daytime hours when automobile traffic peaks. Xiong et al. [27] evaluated that vehicle emissions and mineral dust accounted for 20% and 15% of the total annual PM_2.5_ mass concentrations in Wuhan, respectively. The number of old vehicles, accounting for 7.8% of vehicles on China’s roads, undercut the minimum CAAQS [28]. By the end of 2015, Chongqing, Hubei Province and Jiangsu Province eliminated a total of 6400, 24,800, and 89,900 yellow-label vehicles and outdated registered before the end of 2005 [24]. Although some measures such as government subsidies for purchasing alternative fuel vehicles, levying emission fees for construction-site dust emissions, central heating, relocation of polluting enterprises, water spray along the road to reduce coarse particles from fugitive dust, improving the efficiency of coal burning, have been adopted by the local authorities, our results underscore that more efforts are urgently needed to improve the air quality because current level of particulate matter is still quite high.

**Figure 2:**
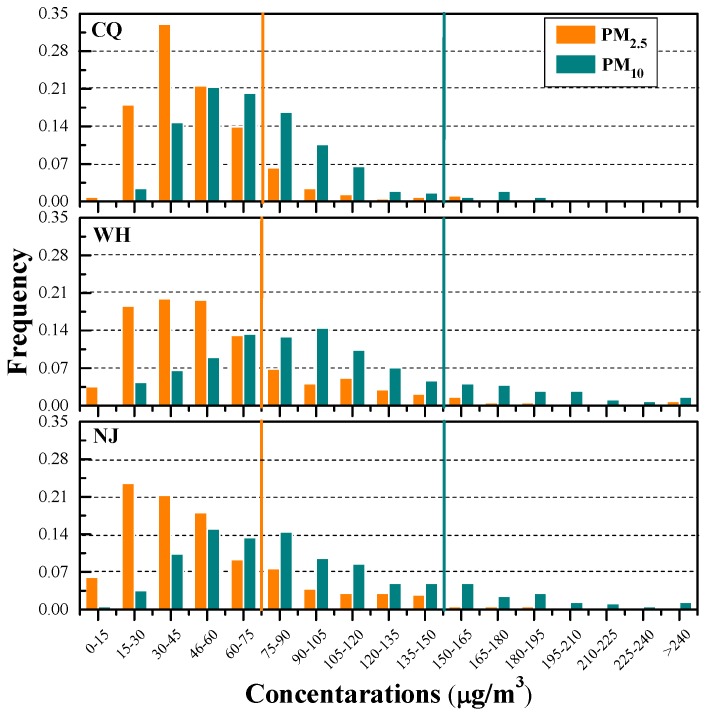
Frequency distributions of daily PM_2.5_ and PM_10_ in Chongqing (CQ), Wuhan (WH), and Nanjing (NJ). Note: the CAAQS-II standards of daily average are plotted as straight lines.

Figure 2 shows the frequency distributions of daily PM_2.5_ and PM_10_ in the three cities. PM_2.5_ concentrations in the range of 15−60 μg/m^3^ dominate most days during studied period, accounting for about 73%, 58% and 63% of all days for Chongqing, Nanjing and Wuhan, respectively. Similarly, PM_10_ concentrations in the range of 30−105 μg/m^3^ dominate most days during the studied period, accounting for approximately 83%, 56% and 63% of all days for Chongqing, Nanjing and Wuhan, respectively. In Chongqing, 13% for PM_2.5_ during the studied period exceeded the corresponding CAAQS-II of concentration limits for 24-h average (75 μg/m^3^), while 4% for PM_10_ exceeded the Grade II daily standards (150 μg/m^3^). As for Wuhan and Nanjing, these percentage values are 25% and 22% for PM_2.5_, and 17% and 15% for PM_10_, respectively. This suggests that these two areas suffer from severe particulate matter pollutions while the situation in Chongqing was slightly better.

O_3_ is one of the major gaseous pollutants in the three cities. Annual average concentrations of O_3_-8 h in Nanjing (49.6 ppb) and Wuhan (43.9 ppb) are approximately 52% and 34% higher than that in Chongqing (32.7 ppb). Although O_3_ annual standards are not defined in CAAQS, daily average O_3_ standards are defined in CAAQS to assess the daily concentrations of O_3_. The frequency distributions of 8 h peak O_3_ in the three cities indicate that 4%, 12%, and 14% in Chongqing, Wuhan, and Nanjing exceeded the CAAQS daily Grade II standards (160 μg/m^3^, 74.7 ppb for 8 h average, and 200 μg/m^3^, 93.3 ppb for hourly average).

**Figure 3:**
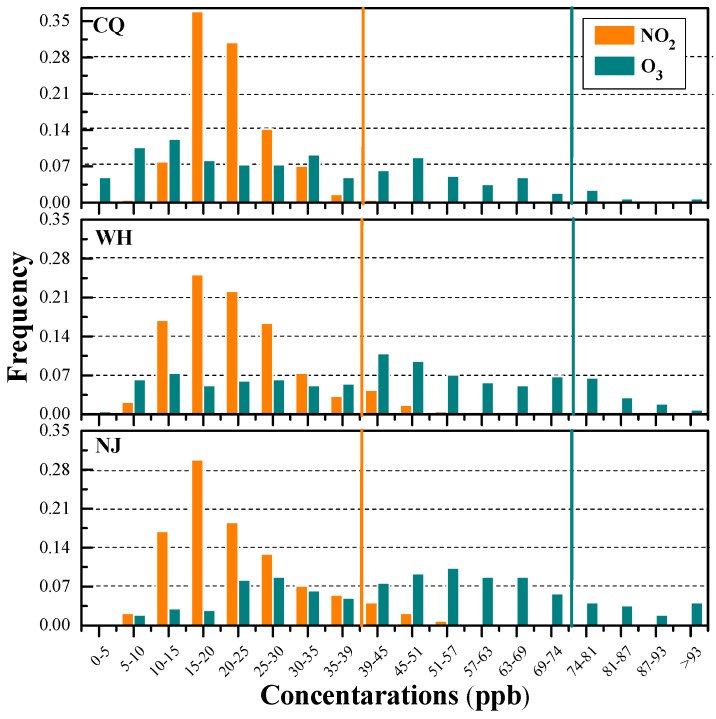
Frequency distributions of daily NO_2_ and O_3_-8 h in Chongqing (CQ), Wuhan (WH), and Nanjing (NJ). Note: the CAAQS-II standards of daily average are plotted as straight lines.

The mean concentrations of NO_2_ are 21.7, 22.7, and 22.9 ppb, with a range of 7.8–46.7, 6.3–57.0, and 7.8–56.0 ppb in Chongqing, Wuhan, and Nanjing, respectively. The annual average NO_2_ concentrations in the three cities are close and all barely reach the Grade II upper limit of annual standards (40 μg/m^3^, 19.5 ppb). Nevertheless, the frequency distributions of daily NO_2_ shown in Figure 3 indicate that about 0.8%, 6.6%, and 7.1% NO_2_ in Chongqing, Wuhan, and Nanjing, respectively, exceeded daily CAAQS-II standard of 80 μg/m^3^ (39.0 ppb).

Sulfur dioxide is a toxic gas with a pungent, irritating smell. The presence of SO_2_ and NO_2_ in the troposphere has significant impacts upon human health, the habitat suitability for plant communities, as well as animal life. SO_2_ averaged as 5.0, 5.1, 6.8 ppb, with a range of 1.8–13.6, 1.4–18.6 and 2.5–17.2 ppb in Chongqing, Wuhan, and Nanjing, respectively. The SO_2_ concentration has steadily decreased, and the annual average NO_2_ concentrations are as large as 3–4 times the SO_2_ concentrations in these hot cities. The annual average SO_2_ concentrations are within the CAAQS-I standards of about 7 ppb (20 μg/m^3^), and daily average concentrations are also within the CAAQS-I (50 μg/m^3^, 17.5 ppb) for Chongqing and Nanjing when those within CAAQS-II (150 μg/m^3^, 52.5 ppb) for Wuhan. According to the Chongqing environmental quality report (2000–2015), the SO_2_ concentrations in urban Chongqing decreased astonishingly from 156 μg/m^3^ in 2000 to 16 μg/m^3^ in 2015, whereas NO_2_ levels changed insignificantly from 54 μg/m^3^ in 2000 to 45 μg/m^3^ in 2015 (http://www.cepb.gov.cn/). Especially, an increasing tendency of NO_2_ emission associated with a huge consumption of petroleum fuels and the expansion of vehicular population was observed in Chongqing since 2011 [26]. The reverse changes in precursors of SO_2_ and NO_2_ led to the downward of SO_4_^2−^ and upward trend of NO_3_^−^. Thus, the (NO_3_^−^)/(SO_4_^2−^) mass ratio of 0.51 in recent study [26] was much higher than that of 0.21 in 2005–2006 [29], comparable to the national average of 0.46, but still lower than Beijing (0.83) and Guangzhou (2.14) [30].

The CO averaged as 0.8 ppm, with a range of 0.4–2.7, 0.4–2.1, and 0.4–1.8 ppm with a low deviation magnitude in Chongqing, Wuhan, and Nanjing, respectively. Like SO_2_, none of the daily average CO concentrations in these cities exceeded the Grade I daily standard of 3.2 ppm (4 mg/m^3^). The emissions of particulate matter via anthropogenic emissions are often accompanied by the emissions of gaseous pollutants (NO_x_, SO_2_ and CO), and the latter is the important precursor for the formation of secondary aerosols, especially fine and ultra-fine particles in the atmosphere.

A substandard day is identified as the day with any pollutant concentration exceeding CAAQS Class II daily standards. As shown in Table 2, Wuhan experienced most severe air pollution with 143 substandard days, and the polluted proportion amounted to 39%; the second worst air quality was Nanjing with 133 substandard days, and the proportion reached to 36%; Chongqing had 64 exceedances in total during the one-year period, and the proportion was 18%, which had the relatively better air quality among the studied cities, while there were still almost 2 months of pollution days.

Overall, for the three second-tier cities, the situation of particle pollution is more serious than the pollution from trace gaseous species. The annual concentrations of particulate matter followed the order of Wuhan > Nanjing > Chongqing, whereas the annual concentration of gas pollutions followed the order of Nanjing > Wuhan > Chongqing. The number of substandard days is gradually increasing from the upper reaches to the middle/lower reaches of the Yangtze River. Recently, Ma et al. [31] and He et al. [32] also used real-time release data on web-platform in three megacities cities, i.e., Beijing, Shanghai, and Guangzhou, to summarize the air pollution situation over eastern China. Compared to their studies, the substandard percentages in Chongqing, Wuhan and Nanjing are comparable to Shanghai, but lower than Beijing. However, the values are higher than Guangzhou.

### 3.2. Seasonal Variations

**Figure 4:**
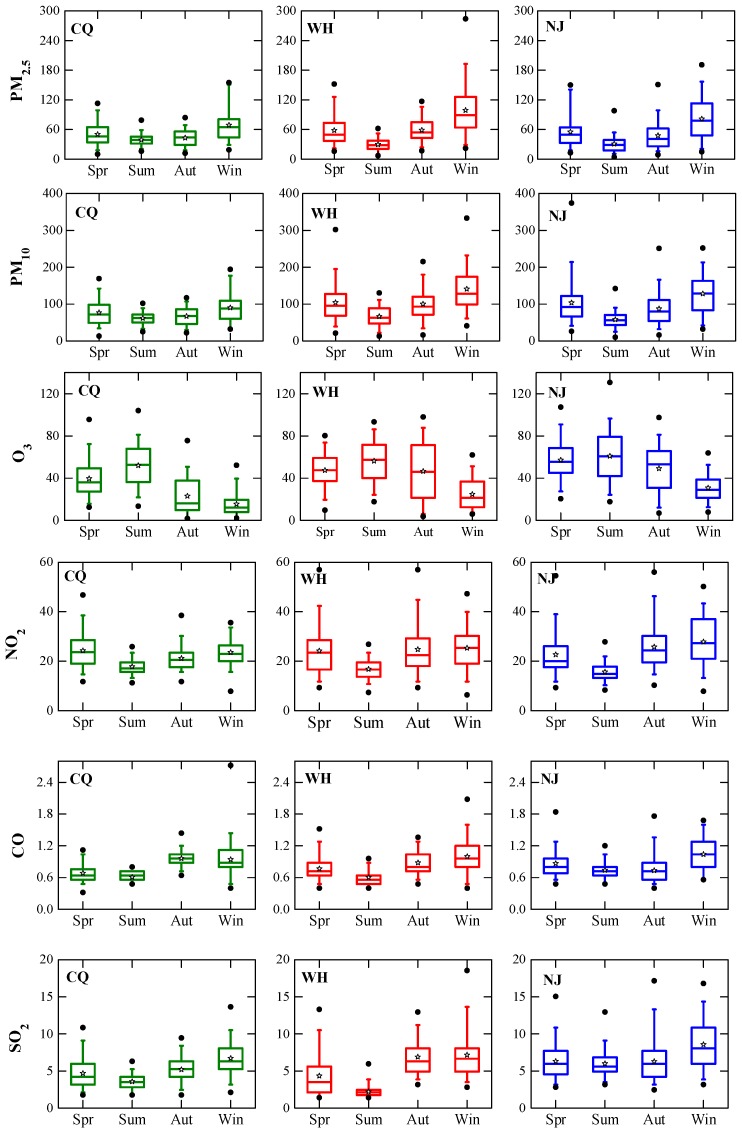
Whiskers box-plots with the concentrations of six air pollutants according to their respective seasons (units are μg/mg^3^ for PM_2.5_ and PM_10_, ppb for O_3_-8 h, NO_2_ and SO_2_, and ppm for CO). The top and bottom whiskers show the 95th and 5th percentile while the upper and lower boundaries of the central box show 75th and 25th percentile. The middle line of the box represents median, pentacle represents the arithmetic average and circle represents max and min values.

The annual analysis in the previous section provides an overview of the air pollutants in the three areas. To make the analysis straightforward, the seasonal distribution (Spring: March–May, Summer: June–August, Autumn: September–November, Winter: December–February) of six pollutant concentrations using the whiskers-box plots which graphically depict numerical data via quartiles based on daily average data are shown in Figure 4. The top and bottom whiskers show the 95th and 5th percentile, while the upper and lower boundaries of the central box show 75th and 25th percentile. The line in the middle of box represents median value, pentacle represents arithmetic average and solid circle represents maximum and minimum values.

Average PM_2.5_ concentrations in Chongqing in all seasons are similar, and the highest concentration occurs in winter (69.0 μg/m^3^). The variances of PM_2.5_ in Wuhan and Nanjing are overall relatively high compared to Chongqing. In Wuhan, average PM_2.5_ concentrations substantially alleviate in summer (29.6 μg/m^3^) compared to average concentrations in other seasons (about 58.5 μg/m^3^ in spring and autumn and 98.8 μg/m^3^ in winter). Seasonal average PM_2.5_ concentrations in Nanjing also peak in winter (81.2 μg/m^3^), followed by spring (55.2 μg/m^3^), autumn (47.9 μg/m^3^), summer (30.8 μg/m^3^), successively. PM_2.5_ accounts for a large fraction of PM_10_ mass in these cities (see below, >50%), thus PM_10_ follows similar seasonal variation trends as PM_2.5_, implying PM_2.5_ control strategies will also impact on reducing the PM_10_ pollution. These results are consistent with previous study of particulate matter seasonal variation across China from 2006 to 2014 [33].

The maximum daily average PM_2.5_ concentrations are 155, 284, and 191 μg/m^3^ for Chongqing, Wuhan and Nanjing, respectively, while the maximum daily average PM_10_ concentrations are 194, 333, and 374 μg/m^3^, respectively. Extreme aerosol pollution events happened in all three cities. PM_2.5_ comprises mainly primary and secondary anthropogenic sources while PM_2.5–10_ is largely produced from both anthropogenic products and natural emissions of re-suspension of local soil and dust storms. Power generation, industrial processes, residential emissions, and transportation are major anthropogenic contributors of particle concentrations in China [34]. Taking into account the high levels, further legislation efforts are expected to suppress particulate matter pollution and improve air quality in these areas, especially during wintertime.

As a rule, the elevated PM_2.5_ concentrations in the winter are possibly attributable to a combination of two aspects. On one hand, local meteorological conditions are considered to affect air quality deterioration; thereby one year ground-level meteorological parameters including temperature, pressure, RH, wind speed, visibility, and precipitation during the studied period are statistically analyzed. As shown in Table 3, unfavorable meteorological conditions for effective air pollution dilution and diffusion during winter-time, such as less precipitation (Chongqing with 118 mm, Wuhan with 87 mm, Nanjing with 107 mm), weaker winds (Chongqing with 1.3 m/s, Wuhan with 1.8 m/s, Nanjing with 2.3 m/s), lower temperature (Chongqing with 9.4 °C, Wuhan with 5.7 °C, Nanjing with 5.5 °C), and higher pressure (Chongqing with 1025.2 hPa, Wuhan with 1027.9 hPa, Nanjing with 1028.2 hPa) compared with those in other seasons contribute to accumulation of PM_2.5_ in winter. Some studies have indicated other meteorological parameters, such as vertical mixing processes (e.g., boundary layer height), atmospheric stability, monsoon, and persistent temperature inversion near surface also affect the formation of high PM_2.5_ pollution events [32,35,36,37]. Quan et al. revealed that the planetary boundary layer (PBL) height and PM_2.5_ concentrations were anti-correlated at Tianjin based on field experiments by three remote sensing instruments (boundary wind profile radar, microwave radiometers, and micro-pulse lidar) [35]. In some severe pollution events, atmospheric chemistry may also play an important role, especially on the effect of the secondary aerosol particles formation [36]. 

Zhang et al. found that the East Asian winter monsoon was abnormally weak, and high pressure inhibited the development of convection [37]. The weakening or static wind was unfavorable for spreading PM_2.5_ to the surrounding areas rapidly, and the decrease of vertical gradient of the horizontal wind reduced atmosphere vertical mixing. Thus, significant inversion in the lower troposphere made the atmospheric stratification more stable, which provided favorable conditions for PM_2.5_ maintenance and accumulation. He et al. [32] pointed out that meteorological condition was the most vital factor for determining the day-to-day variation of pollutant concentrations by affecting pollutant diffusion and deposition in many Chinese cities and contributed more than 70% of the basic day-to-day variations. Atmospheric visibility in winter (4.5–6.4 km) could be significantly reduced due to light extinction by aerosol. High level aerosol loading is generally thought to be responsible for the increased occurrence of haze.

On the other hand, the increasing of energy consumption related to the burning of fossil fuels and biomass leads to the higher PM_2.5_ mass concentrations in the cold season. The coal consumption might be lower currently than decades ago due to the use of new technologies, but industrial and domestic heating is still an important source of coal-fired consumption and biomass burning. Moreover, residential heating emissions are close to the ground level, and are likely to be higher per unit mass of coal than for boilers, therefore, their contribution is of great relevance to winter PM_2.5_ concentrations. Three cities in this study have no household winter heating systems, particle pollution from neighboring provinces using domestic heating should not be ignored. From Figure 4, it is demonstrated that there is a tiny minority of large values in winter, because the mean and median values are below the middle position between max and min, which could result from lasting stagnant weather with calm wind and high RH in winter.

The three cities boast a subtropical humid monsoon climate dominated by hot and humid summer. Compared with winter, the temperature is higher (Chongqing with 29.4 °C, Wuhan with 27.8 °C, Nanjing with 27.4 °C) and atmospheric pressure is lower (Chongqing with 1002.5 hPa, Wuhan with 1005.0 hPa, Nanjing with 1006.1 hPa) in hot summer (Table 3). The more active atmospheric turbulence associated with more frequent physical removal processes such as rain scavenge and wet deposition (Chongqing with 550 mm, Wuhan with 1204 mm, Nanjing with 743 mm), caused by clean air flow from low latitudes and a maritime airstream, are the main driver of relatively clean particulate matter status. Generally, high RH with frequent precipitation corresponds to low PM_2.5_ concentrations due to wet deposition, especially during summer. Otherwise, RH is positively related with PM_2.5_ concentration if no precipitation occurs, presumably attributable to hygroscopic growth of particles, especially in the heavy fog weather during late autumn and winter. These seasonal variations are extensively fluctuating, reflecting weather conditions and emissions. Even though the PM_2.5_ concentrations in summer are the lowest among the four seasons, still the concentrations in Chongqing (39.1 μg/m^3^) outstrip the Grade II standard whereas the concentration in other two cities is under the Grade II standard, probably because minimum amount of total precipitation in Chongqing in summer (550 mm) leads to relatively less washout of particles and thus higher ambient PM_2.5_ concentrations.

As shown in Figure 4, pronounced seasonal variations of O_3_ can be clearly seen, with the maxima occurring in summer (52.1–61.0 ppb), medium in spring and autumn, and minima in winter. Wintertime O_3_ concentrations (15.4–30.8 ppb) are generally much lower than those in other seasons, which shows an opposite seasonal variation from other gas pollutants and particulate matter. In the troposphere, O_3_ is classified as a secondary pollutant due to the fact that it is not directly emitted, but formed through the enhanced photochemical reactions of large amounts of primary precursors emitted from local and adjacent areas. O_3_ mainly has two precursors, volatile organic compounds (VOC), such as alkanes, alkenes, carbonyl compounds, aromatic hydrocarbons, alcohols, organic peroxides, and halogenated organic compounds, and nitrogen oxide emissions (NO_x_), and reactions among them occur in the presence of sunlight [38]. O_3_ concentrations are positively correlated with the intensity of solar radiation. The seasonal variation of O_3_ is likely due to enhanced levels of photochemical products during warm season. The differences between the highest summer and lowest winter mean O_3_ concentrations for Chongqing, Wuhan and Nanjing are 36.7, 31.6, and 30.2 ppb, and it amounts to approximately 112%, 72%, 61% of their annual mean O_3_, implying large seasonal variability, especially for that in Chongqing. Known as the ‘Three Furnaces’ of the Yangtze River Basin, their summers are long and among the hottest in China with abundant heat. Their mean daily temperature in July is up to 32–35 °C, and the maximum can sometimes exceed 38 °C for sustained periods of 10 days. The solar radiation is remarkably associated with air quality as it could be decreased by increased air pollutants via light scattering and absorption of fine particles in the atmosphere during wintertime. In short, particles have the degradation effect on solar radiation. Thus, the reduced sunshine time and reduced temperature in dry winters should be the main reasons of the sharply dropped concentrations of O_3_ under weakened photochemical reactions in the atmosphere. The observed seasonal behavior of O_3_ is different from previous studies conducted in southern China. For example, 14-year of data in Hong Kong gave a climatologically average maximum in autumn and minimum in summer [39]. The dominance of marine air masses, coupled with rainy and unstable weather, leads to low levels of O_3_ in summer. Pollution-laden continental flow from the north, in conjunction with the stable and warm weather in autumn, contributes to the ozone maximum in autumn [39].

The levels of CO exhibit a weak seasonal variation with little change in magnitude. The average CO concentrations in each season are very low, no more than 1.0 ppm. Winter CO concentration level is relatively higher in Wuhan and Nanjing with wider concentration ranges. However, in Chongqing, CO concentration is highest in autumn, and is comparable in the other three seasons. The CO concentration is normally considered as a good index for vehicle emissions. Clearly seen in Figure 4, CO emissions from mobile sources are expected to increase somewhat during the cold season due to cold-starting of vehicles.

The SO_2_ concentrations in Wuhan are highest in winter, followed by autumn and spring. Summer has lowest concentrations (2.2 ppb), only one third of the winter concentration. Furthermore, the interquartile ranges in winter are 4.9–8.0 ppb while they are 1.7–2.3 ppb summer. Similar results can be found in Nanjing and Chongqing but the differences between winter and summer are smaller. High SO_2_ concentrations in winter are consistent with high winter PM_2.5_ and PM_10_ concentrations. Local anthropogenic emissions of SO_2_ come from the burning of fossil fuels containing coal and oil while natural emissions come from volcanic activity. Though SO_2_ concentrations show a strong increase during the wintertime in these cities, it is not significant as the finding in decade ago [26]. This suggests that SO_2_ emissions have been largely reduced by perfect application of desulphurization and effective control of combustion emissions under management of the Chinese government.

The NO_2_ concentrations have a similar distinguishable seasonal pattern as SO_2_ in the three cities. Summer is the best season, with a range of 15.6–17.7 ppb while winter is the worst season with concentrations of 23.5–27.8 ppb. Stagnant weather systems occur more frequently in the cold season, trapping gas pollutants including CO, SO_2_, and NO_x_ near the surface and leading to high concentrations due to the accumulation process. Moreover, the amplitudes of NO_2_ level in winter are greater than in summer. NO_2_ emissions are primarily from traffic vehicles, power plants, and industries with energy exhaust, of which vehicles account for the most. By the end of 2016, the total number of vehicles reached 5.1, 2.3 and 2.4 million for Chongqing, Wuhan, and Nanjing, respectively (Table 1). Some control policies, such as oil upgrading, phasing out of vehicles that fail to meet the European No. 1 standard for exhaust emissions, and traffic restrictions play a certain role in decrease of NO_2_ concentrations. However, in last decade, the drastic growth of traffic vehicles associated with the resulted exhaust emissions have resulted in the sharp increase of NO_x_ and VOCs, leading to a great impact on urban air quality. NO_x_ is nitrate precursor, and vehicle exhaust contains high levels of NO_x_. The efficiency of flue gas desulfurization in coal-fired power plants is obviously higher than the denitrification efficiency, which is another cause of higher concentration of NO_x_ in the atmosphere [40]. Additionally, the concentration of NO_2_ is less affected by regional transport because of its strong chemical activity and short lifetime.

**Figure 5:**
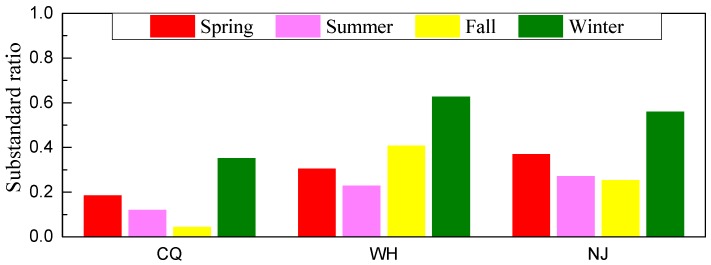
Seasonal variation of substandard ratio in Chongqing (CQ), Wuhan (WH) and Nanjing (NJ).

Figure 5 gives a comprehensive analysis of the air quality evaluations on a seasonal basis under CAAQS-II standards. The air quality was the worst in winter, with substandard percentage of 35% for Chongqing and generally up to 60% for Wuhan and Nanjing. The daily ‘major pollutant’ is identified to measure which pollutant contributes the most to the air quality degradation on substandard days with the basic of AQI system. Daily individual AQI (IAQI) values are calculated using daily concentrations of individual pollutant according to Technical Regulation on Ambient Air Quality (HJ 633-2012) (http://kjs.mep.gov.cn), where the IAQI is 100 when the corresponding pollutant concentration is equal to the CAAQS-II standard. The pollutant that has the maximum AQI is then defined as the major pollutant on that day [41]. PM_2.5_ is typically the most frequent major pollutant to poor air quality with 73%, 64% and 88% accounting for substandard days. Especially in winter substandard days, PM_2.5_ as the major contributor accounts for dominant proportion with 100% for Chongqing, 88% for Wuhan, and 96% for Nanjing, respectively. This is related to two main factors, i.e., emissions and meteorological conditions, as discussed above. Among other seasons, for Chongqing, autumn is the best air quality season with substandard ratio about 4%, while the substandard ratios of summer and spring are higher than 12% and 18%, respectively. On careful analysis it appears that PM_2.5_ as the most frequent major pollutant in autumn and spring and occupies over 65%, while O_3_ pollution is the sole major pollutant in summer under favorable weather conditions with long sunshine hours and high solar radiation. The air qualities for summer and fall are comparable for Nanjing. However, for Wuhan, the substandard ratio is lowest in summer (23%), and air pollution in the fall with a rate over 41% could be an important problem as a result of the biomass burning after harvest time.

Except O_3_, the rest of the five pollutants exhibited almost consistent temporal patterns, with the highest concentrations in winter and the lowest in summer. Under the current CAAQS, PM_2.5_ is the most serious pollutant. With the enhancement of emission control efforts, O_3_ pollution will become increasingly prominent, and challenge the continuous improvement of air quality.

### 3.3. Monthly Variations

**Figure 6:**
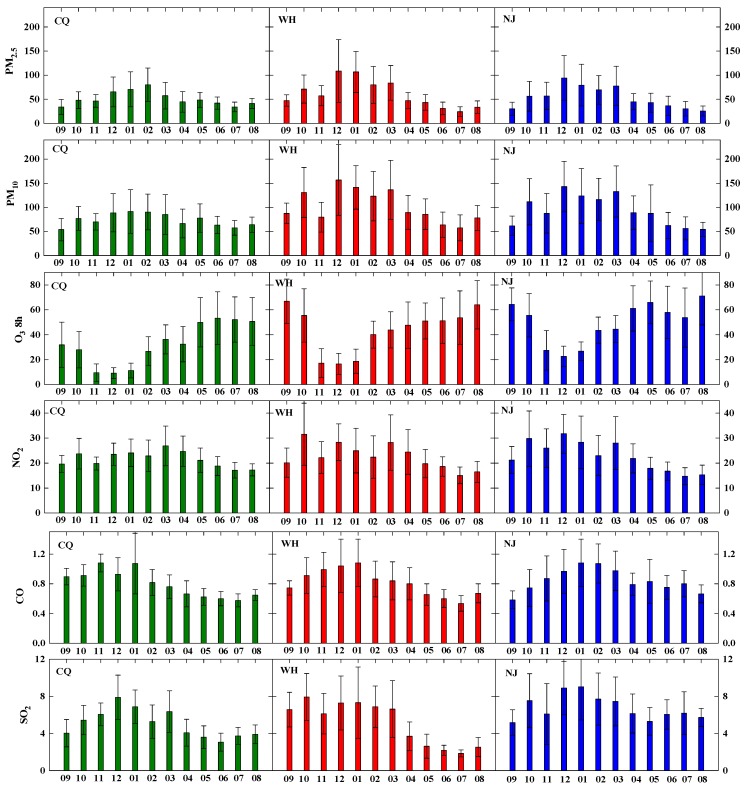
Monthly averaged air pollutants in Chongqing (CQ), Wuhan (WH), and Nanjing (NJ) (The error bar represents standard deviation. Units are μg/mg^3^ for PM_2.5_ and PM_10_, ppb for O_3_-8h, NO_2_ and SO_2_, and ppm for CO).

To learn more about the temporal variations of pollutants concentrations, further comparisons of the six criteria pollutants in month-scale were analyzed (Figure 6). The monthly statistics are calculated using individual daily data, and an almost identical trend and fluctuation during the same period is observed in the three regions. Monthly PM_2.5_ concentrations greatly exceeded the target upper limit as defined by the CAAQS with 35 μg/m^3^, except during July and September in Chongqing, June to August in Wuhan, July to September in Nanjing. Similar to the annual and seasonal variations, the monthly mean of Chongqing is considerably lower as compared with that of other two areas. The coefficient of variation (COV), defined as the standard derivation divided by the mean, is calculated to characterize the temporal changes within a month. The COV values are 26–51%, 25–60%, 37–55% for PM_2.5_, and 25–50%, 24–47%, 27–68% for PM_10_ in three cities during the different months. Many factors (e.g., local primary emission, weather conditions, regional long-range transport), which affect the formation of secondary aerosols, dry/wet deposition and ambient particle pollution levels via complex physical-chemical mechanisms, all play an important role in the month variation.

In April, the heating supply stops as the weather gets warmer, which greatly alleviates anthropogenic emissions. At the same time, deciduous trees begin to germinate after dormancy, which enhances the ability to capture dust. The leaves on the trees with a large waxy surface can intercept atmospheric particles during dry deposition process, and the stomata on the leaf can absorb various gaseous pollutants [42]. In June, July, August, and September, the PM_2.5_ concentrations with lowest standard deviation lighten clearly, below or slightly beyond the CAAQS Grade II standard. The lowest monthly average PM_2.5_ in Chongqing, Wuhan and Nanjing are recorded in July (34 μg/m^3^), July (24.3 μg/m^3^), August (25.7 μg/m^3^), respectively. As result of abundant rainfall with clean airmasses guided by East-Asia monsoons, particles can be wiped off effectively by wet precipitation and good diffusion conditions. During this period, tree growth reaches its peak because of higher temperatures and the availability of sunlight. The ability of trees to capture particles from air is more effective than at any other time, thus the role of urban green spaces should be worth noting. Currently, strengthening urban afforestation has become one of the important approaches to reduce air pollutant concentrations.

The trees begin to tremble defoliation in October and November, and reduce the amount of body water to ready for the winter. Scavenging effects by the absorption of trees give a big discount. It is special that October has higher PM_2.5_ abundances than that of November and September. The elevated PM_2.5_ level in October is likely due to the agricultural open biomass burning activities during the harvest season. This explanation is supported by fire counts map derived from MODIS (not shown) and analysis results with backward trajectory. The importance of biomass burning contributions to PM_2.5_ has also been underscored by previous studies [14,43,44,45]. Based on joint observations in five cities (Shanghai, Hangzhou, Ningbo, Suzhou and Nanjing) of the YRD, the biomass open burning contributed 37% of PM_2.5_, 70% of organic carbon and 61% of elemental carbon, and the impact of open biomass burning was regional, owing to the substantial inter-province transport of airborne pollutants [45]. The PM_2.5_ exposure level could be reduced 47% for the YRD region if complete biomass burning is forbidden and a significant health benefit is expected [45]. The fact is, the total particulate matter emissions from open straw burning in China is about 1.036 billion kg per year [46]. Presently, 0.75 billion Chinese currency has been invested to support the comprehensive utilization and recycling projects of crop straw [47].

The PM_2.5_ concentrations in the studied cities exhibited a sudden rise and remained at a high alert level in December, January, February, and March until the following April, during which time they are much higher than any other month, illustrating the severity to PM_2.5_ pollution during winter and early spring. Many types of vegetation have dropped almost all their leaves to overwinter, thereby reducing the levels of captured particles and absorbance of various gaseous pollutants. Furthermore, the meteorological conditions in wintertime are unfavorable for the dilution of primary pollutants, and enhance the formation of secondary aerosols. For instance, the conversion rates of NO_x_ and SO_2_ from the gas phase to the particle phases of NO_3_^–^ and SO_4_^2–^ were higher [40]. Large increases in coal combustion and the burning of biomass or biofuel for heating, celebrations that include fireworks display in Spring Festival Holiday also contribute to the formation of high PM_2.5_ pollution. The peak events of PM_2.5_ occur in February for Chongqing and December for the other two with monthly total precipitation all less than 50 mm. In Chongqing, the average PM_2.5_ concentration in February is 80 μg/m^3^, with a highest one of 155 μg/m^3^, and in Wuhan and Nanjing, the average PM_2.5_ concentrations in December are 109 and 94 μg/m^3^, with highest ones of 257 and 197 μg/m^3^. The grim situation urgently needs sustainable and effective pollution control strategies for the control of the air quality degradation and its component. By carefully examining Figure 6, PM_2.5_ concentration in March is higher than in February for Wuhan and Nanjing. The two cities are located in the middle and lower reaches of the Yangtze River, where is the famous rice planting bases of China. Organic fertilizer and nitrogen fertilizer are always used to improve soil fertility and provide sufficient conditions for rice growth, so it is possible that the formation of ammonium salts is responsible for the high PM_2.5_ concentration in March, due to the fact that ammonium salts are one of the major components of PM_2.5_ in the two cities and nearby provinces under condition of moderate temperature and high RH during sowing times. As expected, approximatively opposite to the trend of precipitation, variations of PM_10_ monthly mean mass concentration follow a similar convex parabolic pattern as the distribution with PM_2.5_.

The monthly variations of 8 h O_3_ concentrations demonstrate “hook”-shape curves in Chongqing. From May to August, O_3_ concentrations in Chongqing are relatively high (~50 ppb), and an early summer (June) maximum reached 53 ppb, however, the values in other months are relatively low, especially from November to the following January, with a monthly average less than 10 ppb. Wuhan also has a distinguished shape as Chongqing and it remains a high level (~55 ppb) from April to October and a low (~25 ppb) level from November to March, indicating the low pollution levels in late autumn and winter and high levels on both sides. Its highest O_3_ concentration appears in September, the conjecture is that higher temperature, abundant amount of sunshine, and a humid atmosphere help the generation of O_3_. Different from the other two cities, Nanjing has highest O_3_ concentrations with a double-peak configuration, one maximum in late summer (August) and another one in late spring (May). The observed results are similar to what has been reported in previous studies for the YRD region. Available data from either surface measurement at Lin’an [48] or satellite retrievals at Shanghai [49] all suggested two O_3_ peaks (May and September), attributed to a well-defined summertime decrease of O_3_ associated with clean maritime air transported from the Pacific Ocean by the monsoons.

The NO_2_ concentrations perform oppositely to O_3_. NO_2_ and O_3_ concentrations have a strong relationship as both of them are photochemical products. It is likely that high emission of NO_x_, acting as a catalyst, will reduce O_3_ concentrations by titration effects. Three cities have close concentration distribution at each month, and there is a marked increase in the NO_2_ concentrations from mid-autumn to winter. The months with high NO_2_ concentration are October to March (~25 ppb), while those with low NO_2_ are April to September (~15 ppb). The double peak concentrations of NO_2_ in October and March may be caused by the following reasons. Firstly, the surplus phenomenon of crop straws occurred frequently, which caused increase of atmospheric NO_x_ and particle emissions from the direct burning in the field in October. Secondly, atmospheric NH_3_, which is mainly produced by agriculture, can be oxidized to NO_x_, thus increasing NO_2_. The increase of the total sown area with the application of nitrogenous fertilizer, and the development of livestock breeding are also the important factors to aggravate regional NH_3_ and NO_2_ pollution in March.

The changes of CO concentration show strong regularity, with a clear increase trend from June to December, and peaks (~1.0 ppm) at the January of following year before decreasing. SO_2_ concentrations vary similarly to CO and the peak in December or January was ~8 ppb. The slowly rise in SO_2_ and CO in winter months are directly constant with previous discussion.

SO_2_ and CO concentrations in any month are below the Grade-II standards for these cities, whereas the O_3_ and NO_2_ concentrations basically meet the requirements though occasionally they are still high. On the contrary, PM_2.5_ and PM_10_ remarkably exceeded the corresponding standards.

### 3.4. Comparison with Previous Studies

The central government and local authorities have issued a series of control policies to reduce air pollutants in the past few years. Table 4 exhibits six criteria pollutants concentrations to track the historical trends from earlier peer-reviewed researches and this study. The average concentrations from the same time period as provided in previous studies but different year are calculated to facilitate the comparison.

Zhao et al. [50] measured PM_2.5_ in three sampling sites of Chongqing based on one-year long sampling data from March 2005 to February 2006 and the value was 129 μg/m^3^, about 2.5 times than on-line monitoring results in this study. Xie et al. [51] measured gaseous concentrations such as SO_2_, NO_2_, CO, and O_3_ in Nanjing at an urban site and a suburban one during 2008. Compared to our study, SO_2_ was three times as large, NO_2_ was 33% higher, and CO and O_3_ were quite similar. Meanwhile, the annual mean O_3_ concentration was 2.35 ppb lower in downtown than in the suburban area due to higher NO_x_ pollution levels correlated with heavy traffic. A few studies also investigated the high particulate matter pollution formation processes. For example, Kang et al. [52] studied a long-lasting haze episode that occurred from 19 to 31 October 2009 in Nanjing, and pointed out that as a result of the high-pressure system and an extremely small surface pressure gradient, the average daily PM_10_ concentrations were reaching 296 μg/m^3^, approximately three times than the values of the corresponding period in this study.

More studies about particulate matter and gaseous pollutants were performed since 2012, when more attention was paid to the widespread haze-fog events and enactment of Chinese new CAAQS. Inter-annual variations and/or a series of control measures may cause some differences, but the difference is also contributed by data used. Compared with two recent results conducted in Nanjing by research groups who used different sampling devices, measurement methods and choose different sampling sites, the PM_2.5_ mass concentrations of Ding’s report [53] are significantly lower than those of Shen’s report [54], especially in November (111 μg/m^3^ and 146 μg/m^3^) and June (80 μg/m^3^ and 106 μg/m^3^) in the same year. Their observation mass concentrations of particulate matter are roughly equivalent to the estimation given by van Donkelaar et al. [55], which suggested a multi-year average of PM_2.5_ values over 80 μg/m^3^ in eastern China by using satellite data. For Gong’s [56] and Lyu’s reports [57] of Wuhan, the discrepancy is similar, particle concentrations are lower for the former. A more recent study pointed out the average daily PM_2.5_ concentrations in Wuhan were 114.9 μg/m^3^ from 2012 to 2013 [58].

Although the number of environmental monitoring sites is quite different, with 17, 10 and 9 NEM sites including both urban and rural for Chongqing, Wuhan and Nanjing in our study, and only an urban site used in Chen et al. [26] and Shen et al. [54], and three sites used in Xiong et al. [27], the overall inter-annual trend of particulate matter shows a minor decrease in the past 3–4 years, indicating that the general and extreme pollutions have been improved during these years. For example, it is noteworthy that over three year’s effort, relatively lower levels of PM_2.5_ are observed in this study in spring, summer, and autumn when compared to the previous results reported by Chen et al. [26], but winter PM_2.5_ concentrations are quite similar.

He et al. analyzed annual air pollution characteristics during 2014 for 31 provincial capital cities, including Chongqing, Nanjing and Wuhan [32]. The annual average levels of six pollutants for the three cities were 63.5–81.4 μg/m^3^, 95.4–124.9 μg/m^3^, 16.4–27.6 ppb, 18.2–25.9 ppb, 0.7–1.0 ppm, 8.2–11.3 ppb in 2014. The annual mean concentrations decreased 21–28%, 14–25%, 17%–55% for PM_2.5_, PM_10_, SO_2_, respectively, in this study. Other three pollutant concentrations vary differently from city to city: NO_2_ concentrations increased 19% in Chongqing and decreased about 10% for the other two, CO values increased 14% for Nanjing and decreased 11–20% for the other two, and O_3_ values increased 7%, 16% for Chongqing and Nanjing and decreased 6% for Wuhan, which may be driven by the rapid growth of transportation and industry, combined with imperfect control strategies.

The pollutants affect visibility, air quality and human health. Air pollution in China has obviously been reduced in recently years, but it still has a long way to go and big challenges in air quality improvement remain. More detailed comparisons need to be carried out in the future to reach more robust conclusions, and quantitative studies and exploration of the relationships between atmospheric environment and urbanization can provide references for sustainable urban development planning and environmental policy formulation.

### 3.5. Episode Days

Sources of particulate matter include not only the direct emission of primary mineral particles, but also secondary anthropogenic particles from homogeneous and heterogeneous reaction with gaseous precursors based on source apportionments. Huang et al. [36] investigated the chemical components during severe pollution periods in Beijing, Shanghai, and Guangzhou in China, and pointed out that 50–75% of PM_2.5_ was contributed by secondary aerosol in these eastern cities. Particle size is a key parameter determining the impact on human health. Actually, fine particles get more attention because of their smaller size, longer lifetime in air, and greater health risks than coarse particles.

Since fine-mode and coarse-mode particles come from diverse sources and perform different physic-chemical properties, the PM_2.5_ to PM_10_ ratios (PM_2.5_/PM_10_) can act as an indicator to reveal characteristics of particle pollution and characterize the underlying atmospheric processes in absence of direct measurement [14,37,59]. The results of ratios have business with local emission sources, climatic, geographic and geological conditions. A higher ratio attributes particle pollution to anthropogenic products while a smaller ratio indicates substantial participation of coarse particles such as dust storms. Annual average PM_2.5_/PM_10_ ratios are about 0.67, 0.58, and 0.55 in Chongqing, Wuhan, and Nanjing, respectively. In other words, PM_2.5_ contributes substantial fraction of PM_10_ (67%, 58% and 55%) in the above cities, indicating the dominant fraction of fine particles in PM_10_. The unique valley terrain in Chongqing might be responsible for the formation of secondary aerosols, one of the major sources of fine PM_2.5_ particles, resulting its relatively higher annual mean ratio. It is very clearly that these ratios are significantly higher than those found in Northwest and West China (0.38–0.52), where local primary mineral dust emission and desert-like regional dust transport from dry and bare land make a great contribution. Other coarse-mode particle such as biogenic-derived large particles, such as plant debris, could have a minor if not a negligible contribution with its cold desert climate and low vegetation coverage [14].

PM_2.5_ episode days mean daily average PM_2.5_ > 75 μg/m^3^. The PM_2.5_ concentrations on the episode and non-episode days show quite close to each other in these cities. Average PM_2.5_ concentrations during the episode days are 100, 118, and 110 μg/m^3^ for Chongqing, Wuhan, and Nanjing, 2–3 times higher as compared to PM_2.5_ concentrations during the non-episode days which were 43, 42, and 38 μg/m^3^, respectively (Table 5). The number of episode days in Wuhan was approximately 1.2 times greater than in Nanjing, and 2.0 times than in Chongqing. The annual average PM_2.5_/PM_10_ ratios increased from 0.66 on non-episode days to 0.76 on episode days in the Chongqing, and increased from 0.54 on non-episode days to 0.73 on episode days in Wuhan, and increased from 0.52 on non-episode days to 0.66 on episode days in Nanjing. Large differences of PM_2.5_/PM_10_ ratios between episode and non-episode days are found in Wuhan (0.19), which are almost twice as frequent as in Chongqing (0.10) due to lesser number of episode days in the latter. Average PM_2.5_ concentrations in episode days over 100 μg/m^3^ are rarely reported, and meanwhile, the PM_2.5_/PM_10_ ratios on episode days in this study are higher than non-episode days, suggesting significant production of secondary aerosol on episode days.

**Figure 7:**
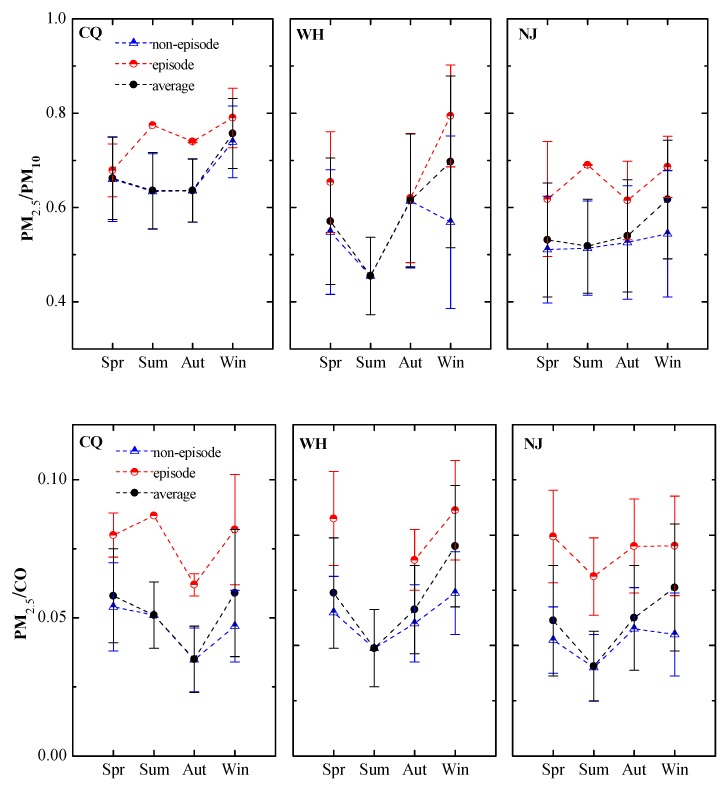
Seasonal ratios of PM_2.5_/PM_10_ (**up**) and PM_2.5_/CO (**down**) in the three cities.

Figure 7 show seasonal ratios of PM_2.5_ to PM_10_ during the whole study period, PM_2.5_ episode, and non-episodes days in three cities. Notably, no episode days are observed in July, August and September in all three cities, which is related to reduced anthropogenic emissions and better weather conditions (Figure S1). The episode days occur from mid-fall to winter and then to spring. Consistent with the periodical variation of PM_2.5_ concentrations, the PM_2.5_/PM_10_ ratio reaches the maximum in wintertime and it drops to a comparatively low level in summer. The stable atmospheric conditions are beneficial for more secondary aerosol accumulation, also, favor the dry deposition of coarse particles, thus making the fine particles dominant in PM_10_ in winter ultimately. On the contrary, the most polluted season for some cities located in Northwest and West China appears in spring but not in winter. In China, dust storms commonly originate from two regions: the Gobi Desert that lies within Mongolia and northern China, and the Taklimakan Desert in western China. The PM_2.5_/PM_10_ ratio during the spring in Korla, one of most frequent occurrence of dust events such as floating dust, dust storm, and blowing dust in West China, was observed as low as 0.21, suggesting the fine particles might not be the major factor contributing to the increase in PM_10_ in that case [14].

To qualitatively infer the contribution of secondary production to PM_2.5_, the ratios of PM_2.5_ to CO are also calculated based on the fact that CO is an excellent tracer and can be used to normalize PM_2.5_ to exclude the influence of primary combustion sources and meteorological factors [14,37]. As shown in Figure 7, PM_2.5_/CO ratios vary pronouncedly among episode (0.077–0.085) and non-episodes (0.040–0.048) days, suggesting the different secondary/primary contributions. Similar to the aforementioned PM_2.5_/PM_10_ ratios, relative larger values of the PM_2.5_/CO ratio are also observed during winter than during the other seasons, which further demonstrates the temporal variation of the contribution of secondary components to PM_2.5_ mass from another point of view. As a comparison, the corresponding values were low in northwest Chinese cities located in downstream of Taklimakan and Gobi Deserts, implying a smaller contribution of secondary aerosols to PM_2.5_ mass [32].

### 3.6. Back-Trajectory

**Figure 8:**
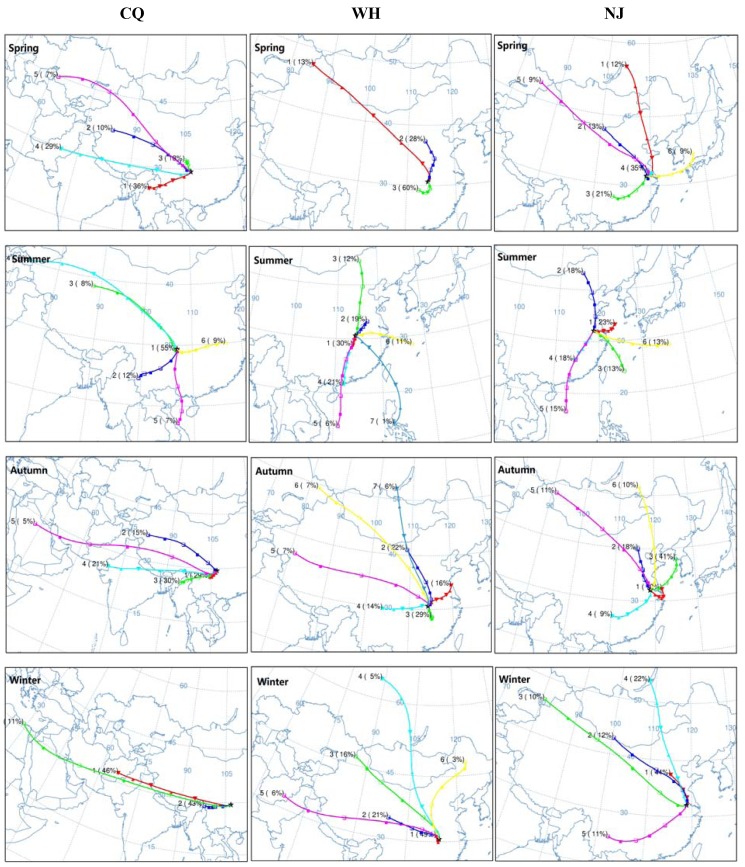
72 h airmass backward trajectories analysis for seasonal distribution in Chongqing, Wuhan, and Nanjing using hierarchical clustering method.

The distribution of anthropogenic emissions of particulate matter is rather uneven. Both local and remote sources account for particle pollution, to determine its sources and assess the impact of long distance transport of airmasses on the air quality, 72 h back-trajectory analysis of airmasses based on seasonal distributions for three cities are conducted during studied period (Figure 8).

The prevailing wind fields for the three regions connect with the local particulate matter concentrations. Under the influence of the East Asian summer monsoon from the tropics, prejudiced south wind is the prevailing wind in the Yangtze River Basin. The airmasses mainly originate from the South China Sea and Northwest Pacific Basin, and then move through southern China prior to reaching the destination. The time from mid-June to the end of July is the ‘meiyu’ season, during which these cities experience a period of mild rain as well as dampness. Associated with enhanced convection within a higher atmospheric mixing layer, the particle minima observed in summer in the three cities could be partially ascribed to the strong dilution effect through abundant wet depositions. However, in winter, the airmasses generally originate from the inner Eurasian continent, causing a high residence time from Mongolia to the North China Plain. Associated with a strong continental high pressure system, those continental airmasses in winter are generally transported from north to south part of China, directly sweeping over the Yangtze River Basin.

Chongqing is located on the southeast of the Sichuan Basin, and surrounded by mountains on the north, east, and south sides and the Tibetan plateau to the west. The transition of mountain-valley breezes during morning and evening can modulate pollutant concentrations in the atmosphere. Atmospheric environment gets better in summer with hot and rainy weather. The mass concentrations of particulate matter are potentially influenced by both autochthonous and input particles. The west, northwest and southwest winds from the mid-west of the Sichuan Basin is slightly more frequent in Chongqing during spring, autumn and winter as a consequence of its distinctive geographical pattern. Therefore, air pollutant concentrations are significantly affected by regional transport under the domination of prejudiced west winds. Back-trajectories confirm the dominance of western deserts for aerosol pollution in Chongqing and suggest two transport pathways. One pathway is traced to the western Gobi Desert. As cold air outbreaks from the northwest, particulate matter is strongly mobilized by the cold front between a high pressure system over Siberia and low pressure system over Mongolia, and subsequently transported to southeastward Chongqing [60]. Another pathway generally originates from the Takla Makan Desert. Under the special terrain-effect of its basin and the surrounding mountains, the airmasses are lifted rapidly by high wind speeds and merged quickly into the conveyer belt between the Takla Makan and the Tibetan by the prevailing westerlies [50].

As for Wuhan, there are many lakes and mountains in this area, resulting in the following meteorological characteristics: higher RH, low wind velocity, frequent fog and haze, poor visual situation. Due to these topographic factors, Wuhan becomes a passage for cold air coming down from the north and west in winter, when weather changes are fierce. By examining the histories of air masses, short distance transport (49%) means local airflow is moving slowly, which is not conducive to the diffusion of air pollutants, dominatingly leading to the highest particulate matter concentrations in winter. Except for a local source, the transportation of neighboring provinces could not be neglected. Mountain breezes from remote areas with long distance transport, such as north wind (northwest, north, and northeast winds) followed by west wind, further strengthen the effect of particulate matter pollutants sinking into Wuhan. This is because northerly wind always transports relatively dirty air from more polluted provinces, such as Henan Province, Hebei Province and Shanxi Province, contributing to air pollution episodes. Additionally, radiation inversion usually occurs in the morning and night, and is unfavorable to air diffusion, which further aggravates particulate matter pollution in winter. Dust storms occur frequently in northern and northwestern China and lead to higher aerosol concentrations in Wuhan, which could cause raised pollution during dry days in spring. In summer, the region is controlled by the southwest monsoon (57%), leading to the best condition for the dispersion of air pollution and summer average of PM_2.5_ concentrations are below the CAAQS-II standards ultimately. In autumn, airmasses from all directions, including east (16%), north (35%), west (21%) and south (29%), respectively, have an impact on the particle concentrations in Wuhan. Pollutant input from industrial cities around Wuhan with intensive coal combustion should be responsible for the increasing of particulate matter concentration. For example, the cities which are famous for smelting, namely Huangshi and Daye, are to the east of Wuhan, and exogenous pollutants from the east easily cause the severe air pollution.

In Nanjing, the north wind is also the prevailing wind in winter, accounting for more than 80% of total winds, indicating the long-range transportation of air pollutants from north polluted region, e.g., the Beijing-Tianjin-Hebei, Shandong Province, and north of Jiangsu Province, is highly related to the worsening air condition with severe and long duration pollution events. Effects of inter-regional transport were also found by studies using the source-oriented Community Multiscale Air Quality (CMAQ) model [61]. Moreover, air pollution situation is also grim in Nanjing under the control of east wind with a great quantity of flourishing industries and vehicle emissions from the southeastern part of Jiangsu Province, the northern part of Zhejiang Province, and megacity Shanghai, especially during autumn. Spring is the transitional period between winter monsoon and summer monsoon. There is alternate current of cold and warm, and the activities of cyclones and fronts are frequent. Intrinsic factors associated with formation of secondary aerosol and dust propagation from north China to South China exacerbate the complex air pollution in spring. Nanjing is far from desert region, however, its air quality can still be affected by spring outbreak of dust storms under certain conditions. Based on Back-trajectory analysis, on the day of 7 May 2016, regional dust originated from northern China, and then gone through Henan Province and Shandong Province, ultimately penetrated into Nanjing, leading to occurrence of high PM_10_ concentration with 374 μg/m^3^ under the domination of dry and strong northern winds.

The back-trajectory results demonstrate that improving air quality is not a merely local issue, but a regional issue. Currently in China air pollution control measures are implemented relatively independently by the local governments. It is a challenge for policy makers to establish collaborative control measures across administrative borders.

### 3.7. Correlations between Air Pollutants

To establish the relationship between different pollutants, Pearson correlation coefficients (R) are calculated during the study periods and the corresponding results are shown in Table 6, ranging from 0.50–1.00 (strong positive correlation), 0.25–0.49 (moderate positive correlation), 0–0.24 (weak positive correlation), −0.25 to −0.01 (weak negative correlation), −0.50 to −0.26 (moderate negative correlation).

It can be seen that there are some analogies between the three cities. PM_2.5_ has a very strong positive relationship (R: 0.96 for Chongqing, 0.88 for Wuhan, 0.92 for Nanjing) with PM_10_ over the one-year period possibly on account of higher PM_2.5_/PM_10_ radio of the study areas (Figure 7), indicating these species are normally emitted from identical sources and/or that they are influenced by similar meteorological conditions. The correlation coefficients of the linear regression model between particulate matter and SO_2_ and NO_2_ are also significantly high (PM_2.5_ with SO_2_: R = 0.58–0.63, PM_10_ with SO_2_: R = 0.63–0.66, PM_2.5_ with NO_2_: R = 0.57–0.68, PM_10_ with SO_2_: R = 0.68–0.73). Particulate matter is actually a complex mixture of extremely small particles and droplets, and contains organic and element carbon, ions (e.g., sulfate, nitrate, and ammonium), and heavy metals (As, Mn, Cr, Ni, Cd, and Hg). NO_2_ and SO_2_ often have strong positive correlations with particulate matter because SO_4_^2−^ and NO_3_^−^, which are emitted directly together with NO_2_ and SO_2_, or as secondary inorganic aerosol (SIA) generated via oxidation process, could further enhance fine PM_2.5_ abundance [36]. NO_2_ and SO_2_ themselves are also strongly correlated (R = 0.60–0.72), implying that they have mutual origin and elimination reactions.

In the presence of sunlight, these species react chemically with each other, producing toxic secondary pollutants such as O_3_. The detailed mechanisms still need to be further addressed through long-term measurement of aerosol chemical composition. It is encouraging to notice the correlation between CO and PM_2.5_ (0.52–0.81) is slightly higher than that between CO and PM_10_ (0.48–0.70), thus suggesting that CO emission process is accompanied by the emission of fine particles. However, O_3_ concentrations become moderate or weak negative correlation with particulate matter (O_3_ with PM_2.5_: R = −0.11 to −0.26, O_3_ with PM_10_: 0.03 to −0.14) and other gaseous pollutants (O_3_ with SO_2_: R = −0.07 to −0.30, O_3_ with NO_2_: 0.00 to −0.27, O_3_ with CO: −0.23 to −0.46). It is partially because O_3_ is a secondary formation product and easily depletes during the oxidation of freshly emitted NO to NO_2_, particularly in cold seasons and at nighttime.

The correlation degrees among pollutants vary largely in different seasons, indicating the meteorological factors in each season influence the atmospheric pollution. In spring, all six pollutants except for O_3_ are significantly correlated with each other. In Wuhan and Nanjing, O_3_ has weak correlations with SO_2_, NO_2_, CO, PM_2.5_ and PM_10_. In Chongqing, O_3_ is also weakly correlated with PM_2.5_, NO_2_ and CO, whereas the main difference from the other two cities is that O_3_ is moderately negatively correlated with SO_2_ while positively correlated with PM_10_. In summer, when O_3_ concentrations are higher, a moderately positive (0.5 > R ≥ 0.25) or even strongly positive (R > 0.5) with other pollutants has been often observed, especially in Wuhan. It reflects the simultaneous formation of O_3_ and secondary fine particles facilitated by the enhanced VOCs/NO_x_ emissions through strengthened photochemical reactions, due to the stronger solar radiation in sunny weather. All other pollutants are still interrelated, but the correlations are lower than other three seasons. The correlations in the autumn are principally similar to those in the spring. However, as compared to spring, CO correlations with PM_2.5_, PM_10_, SO_2_ and NO_2_ become remarkably lower during autumn in Chongqing and Wuhan. Highly reverse correlations are observed between O_3_ and CO in Chongqing. High-pressure system during winter leads to stagnant weather conditions near the surface with weak wind, relatively low boundary layer height, and shallow mixing layer. Under this condition, the vertical mixing of precursors and the dispersion of pollutants are very limited. More primary pollutants could inhibit formation of secondary pollutants such as O_3_ through scattering or absorbing solar radiation [15,62,63]. The O_3_ has weak (−0.25 ≤ R < 0) or moderate (−0.5 ≤ R < −0.25) negative correlations with other five pollutants due to the fact that particulate matter and other gas pollutions are prone to formation, processing, and accumulation whereas the formation of O_3_ is limited by weaker sunlight with high aerosol pollution in colder season.

## 4. Conclusions

We used recently released ambient monitoring data in three metropolises, i.e., Chongqing, Wuhan, and Nanjing, to evaluate the current situations of particulate matter and trace gases for the Yangtze River Basin. The annual average concentrations of particulate matter followed the order of Wuhan > Nanjing > Chongqing, while the annual concentration of gaseous pollutants followed the order of Nanjing > Wuhan > Chongqing. About 13%, 25%, 22% for PM_2.5_, and 4%, 17%, 15% for PM_10_ in Chongqing, Wuhan and Nanjing, respectively, exceeded CAAQS Grade II daily standards. By comparison, SO_2_ and CO concentrations were consistently below Grade-II standards but NO_2_ concentrations faintly above Grade-II standards with 1–7%, especially in March and October. Distinct seasonal trends were observed for PM_2.5_, PM_10_, CO, SO_2_, and NO_2_ with the maximum in winter and the minimum in the summer while O_3_ exhibited exactly an opposite pattern. Possible impact of meteorological conditions on ambient pollutant concentrations was investigated to understand inter-seasonal and monthly variability. PM_2.5_ was the predominant contributor to the poor air quality during the studied period and O_3_ pollution was significant contributor in summer. The values of PM_2.5_/PM_10_ and PM_2.5_/CO indicate that the formation of secondary aerosols plays an important role in the aggravation of air pollution during haze episode days. Air pollutant concentrations are also significantly affected by regional transport under the domination of the prejudiced winds with the back-trajectory hierarchical clustering analysis, thus both local and extra-regional environment pollution synchronous control and joint prevention should be implemented. Additionally, moderate to strong correlations between pollutants except O_3_ were found in most seasons, whereas O_3_ was roughly positively related with other pollutants in summer but negatively related in other seasons. The air quality of these famous second-tier cities is up to Shanghai, better than Beijing, and worse than Guangzhou. Future research will be conducted to further obtain a more comprehensive understanding during the highly polluted seasons, in order to elucidate the underlying mechanisms responsible for the formation of air pollution and to provide necessary scientific guidelines for policy makers in their risk management of urban air pollution and better urban governance around the Yangtze River Basin.

## Figures and Tables

**Table 1:** Basic Information of the three studied cities by the end of 2016. Source: statistical bulletin on national economic and social development, cited from the web of http://www.stats.gov.cn/tjsj/.

City	Number of NEM Sites	Latitude (Degree)	Longitude (Degree)	Altitude (m)	Area (km^2^)	Population (million)	Death Rate (‰)	GDP (billion)	Vehicle Counts (million)
Chongqing	17	29.4	106.9	259.1	82,400	30.5	7.2	1755.9	5.1
Wuhan	10	30.6	114.3	23.3	8494	10.8	5.4	1191.3	2.3
Nanjing	9	32.1	118.8	8.9	6587	8.3	5.7	1050.3	2.4

**Table 2:** Annual mean concentrations of six criteria pollutants in Chongqing, Wuhan, and Nanjing during 2015–2016 ^a^.

City	PM_2.5_ (μg/m^3^)	PM_10_ (μg/m^3^)	O_3_-8 h (ppb)	NO_2_ (ppb)	CO (ppm)	SO_2_ (ppb)	Substandard Ratio ^b^
Chongqing	50.3 ± 25.3	73.9 ± 32.0	32.7 ± 21.7	21.7 ± 5.7	0.8 ± 0.2	5.0 ± 2.1	17.5%
Wuhan	61.3 ± 40.9	102.7 ± 53.3	43.9 ± 23.2	22.7 ± 9.0	0.8 ± 0.3	5.1 ± 3.1	39.1%
Nanjing	53.7 ± 35.6	94.0 ± 51.4	49.6 ± 22.7	22.9 ± 9.2	0.8 ± 0.3	6.8 ± 2.7	36.3%

^a^ The error denotes one standard deviation. ^b^ The percentage of days with any pollutant concentration exceeding Grade II standards during the studied period.

**Table 3:** Annual and seasonal mean values with standard deviations of meteorological parameters in Chongqing, Wuhan, and Nanjing, respectively.

	Temperature (°C)	Sea-Level Pressure (hPa)	Relative Humidity (%)	Wind Speed (m/s)	Visibility (km)	Total Precipitation (mm)/Number of Rainy Day
*Annual*						
Chongqing	19.7 ± 8.3	1013.7 ± 10.0	74.7 ± 15.6	1.4 ± 0.7	7.7 ± 6.0	1528/228
Wuhan	17.3 ± 9.6	1016.4 ± 10.0	79.3 ± 19.5	1.7 ± 1.4	8.5 ± 6.8	1931/196
Nanjing	16.8 ± 9.5	1017.2 ± 9.6	71.4 ± 18.3	2.5 ± 1.5	5.3 ± 3.5	1427/163
*Spring*						
Chongqing	19.6 ± 4.7	1011.4 ± 6.4	73.5 ± 16.7	1.4 ± 0.8	7.0 ± 5.2	384/61
Wuhan	17.6 ± 5.7	1014.2 ± 6.3	77.8 ± 19.9	1.9 ± 1.5	8.0 ± 6.4	317/55
Nanjing	16.3 ± 5.6	1015.6 ± 6.4	69.3 ± 18.6	2.8 ± 1.7	5.0 ± 3.3	334/43
*Summer*						
Chongqing	29.4 ± 4.6	1002.5 ± 4.0	68.0 ± 16.6	1.6 ± 0.8	12.4 ± 6.4	550/62
Wuhan	27.8 ± 4.6	1005.0 ± 3.2	80.0 ± 16.0	1.8 ± 1.4	12.4 ± 8.1	1204/50
Nanjing	27.4 ± 4.5	1006.1 ± 2.9	75.9 ± 15.6	2.4 ± 1.3	6.5 ± 4.0	743/41
*Autumn*						
Chongqing	20.5 ± 4.4	1015.5 ± 5.6	79.1 ± 12.7	1.4 ± 0.7	6. 5 ± 5.1	476/56
Wuhan	17.8 ± 6.9	1018.6 ± 6.0	82.9 ± 19.1	1.5 ± 1.3	7.2 ± 5.2	323/49
Nanjing	18.0 ± 6.3	1019.4 ± 5.9	74.0 ± 17.0	2.5 ± 1.4	5.2 ± 3.3	243/43
*Winter*						
Chongqing	9.4 ± 3.3	1025.2 ± 6.8	78.2 ± 13.5	1.3 ± 0.7	4.5 ± 3.4	118/49
Wuhan	5.7 ± 4.9	1027.9 ± 6.3	76.7 ± 22.2	1.8 ± 1.4	6.4 ± 5.7	87/42
Nanjing	5.5 ± 5.0	1028.2 ± 6.0	66.5 ± 20.2	2.3 ± 1.5	4.5 ± 3.1	107/36

**Table 4:** Comparisons of air pollutants in three cities with other studies. Note here 1 h average concentrations of O_3_ were used.

PM_2.5_ μg/m^3^	PM_10_ μg/m^3^	O_3_ ppb	NO_2_ ppb	CO ppm	SO_2_ ppb	Sampling Period	Site	Reference
129.0						March 2005–February 2006	Chongqing	[50]
50.3						September 2015–August 2016		this study
		28.4	30.4	1.1	18.6	January–December 2008	Nanjing	[51]
		30.7	22.9	0.9	6.8	September 2015–August 2016		this study
	296					19–31 October 2009	Nanjing	[52]
	109					19–31 October 2015		this study
76		19		0.7	10	August 2011–July 2012	Nanjing	[53]
54		31		0.8	7	September 2015–August 2016		this study
146	193					November 2011	Nanjing	[54]
57	88					November 2015		this study
89	138					March 2012		
77	133					March 2016		
106	145					June 2012		
37	62					June 2016		
86	113					August 2012		
26	54					August 2016		
52	71					May–June 2014	Wuhan	[56]
81.2	135.1							[57]
37.3	73.8					May–June 2016		this study
78	94					October–November 2014		[56]
85.3	118.9							[57]
64.2	105.3					October–November 2016		this study
114.9						August 2012–July 2013	Wuhan	[58]
61.2						September 2015–August 2016		this study
57.3	88.2					June–August 2012	Chongqing	[26]
39.1	61.6					June–August 2016		this study
64.9	87.5					September–November 2012		
43	67.1					September–November 2015		
68.0	91.7					December 2015–February 2013		
69	90.2					December 2015–February 2016		
90.1	121.8					March–May 2013		
50.2	76.7					March–May 2016		
141						10–23 October 2011	Wuhan	[27]
86						10–23 October 2015		this study
173						2–15 January 2012		
124						2–15 January 2016		
113						2–15 April 2012		
55						2–15 April 2016		
88						2–15 June 2012		
41						2–15 June 2016		
63.5	95.4	16.4	18.2	0.9	8.3	January–December 2014	Chongqing	[32]
50.3	73.9	17.6	21.7	0.8	5.0	September 2015–August 2016		this study
81.4	119.1	27.6	25.9	1.0	11.3		Wuhan	
61.2	102.7	25.9	22.7	0.8	5.1			
74.7	124.9	26.4	25.0	0.7	8.2		Nanjing	
53.7	94.0	30.7	22.9	0.8	6.8			

**Table 5:** Annual mean values with standard deviations of PM_2.5_/PM_10_ and PM_2.5_/CO in Chongqing, Wuhan, and Nanjing.

	PM_2.5_ Concentrations (μg/m^3^)			PM_2.5_/PM_10_			PM_2.5_/CO		
	Episode Days	Non-Episode Days	Whole Days	Episode Days	Non-Episode Days	Whole Days	Episode Days	Non-Episode Days	Whole Days
Chongqing	100	43	50	0.76 ± 0.08	0.66 ± 0.09	0.67 ± 0.09	0.081 ± 0.017	0.047 ± 0.015	0.051 ± 0.019
Wuhan	118	42	61	0.73 ± 0.14	0.54 ± 0.14	0.58 ± 0.16	0.085 ± 0.018	0.048 ± 0.015	0.052 ± 0.022
Nanjing	110	38	54	0.66 ± 0.09	0.52 ± 0.11	0.55 ± 0.12	0.077 ± 0.017	0.040 ± 0.014	0.048 ± 0.021

**Table 6:** Pearson correlation coefficients of air pollutants in the three cities based on annual and seasonal data in 2015–2016. Different colors represent different ranges: light blue (R ≥ 0.5, strong positive correlation), green (0.5 > R ≥ 0.25, moderate positive correlation), yellow (0.25 > R ≥ −0.25, weak correlation), red (−0.25 ≥ R ≥ −0.5, moderate negative correlation).

	Chongqing					Wuhan					Nanjing				
	PM_10_	SO_2_	NO_2_	CO	O_3_-8 h	PM_10_	SO_2_	NO_2_	CO	O_3_-8 h	PM_10_	SO_2_	NO_2_	CO	O_3_-8 h
annual															
PM_2.5_	0.96	0.63	0.65	0.52	−0.11	0.88	0.58	0.57	0.81	−0.26	0.92	0.60	0.68	0.76	−0.23
PM_10_	–	0.66	0.73	0.48	0.03	–	0.63	0.68	0.70	−0.01	–	0.65	0.71	0.68	−0.14
SO_2_		–	0.60	0.54	−0.30		–	0.72	0.58	−0.11		–	0.70	0.62	−0.07
NO_2_			–	0.44	−0.03			–	0.59	0.00			–	0.60	−0.27
CO				–	−0.46				–	−0.33				–	−0.23
Spring															
PM_2.5_	0.96	0.61	0.78	0.77	0.19	0.89	0.76	0.67	0.75	−0.06	0.84	0.48	0.72	0.71	−0.21
PM_10_	–	0.66	0.79	0.73	0.32	–	0.76	0.69	0.69	0.14	–	0.53	0.64	0.51	−0.08
SO_2_		–	0.69	0.67	−0.26		–	0.71	0.62	0.02		–	0.75	0.51	0.01
NO_2_			–	0.83	0.08			–	0.72	0.05			–	0.66	−0.21
CO				–	−0.05				–	−0.21				–	−0.17
Summer															
PM_2.5_	0.91	0.13	0.56	0.61	0.37	0.90	0.31	0.53	0.64	0.69	0.94	0.30	0.43	0.67	0.32
PM_10_	–	0.44	0.67	0.48	0.56	–	0.46	0.52	0.51	0.81	–	0.45	0.41	0.62	0.48
SO_2_		–	0.22	−0.06	0.44		–	0.39	0.19	0.51		–	0.20	0.41	0.39
NO_2_			–	0.50	0.47			–	0.38	0.36			–	0.49	−0.01
CO				–	0.22				–	0.36				–	0.11
Fall															
PM_2.5_	0.96	0.58	0.57	0.46	0.19	0.86	0.57	0.73	0.58	0.19	0.93	0.65	0.58	0.76	0.13
PM_10_	–	0.67	0.69	0.40	0.31	–	0.78	0.88	0.43	0.50	–	0.78	0.74	0.69	0.26
SO_2_		–	0.53	0.38	−0.07		–	0.81	0.26	0.45		–	0.83	0.62	0.20
NO_2_			–	0.20	0.40			–	0.45	0.35			–	0.58	0.11
CO				–	−0.30				–	−0.21				–	−0.14
Winter															
PM_2.5_	0.98	0.57	0.63	0.49	0.07	0.84	0.21	0.39	0.85	−0.15	0.93	0.54	0.63	0.72	−0.14
PM_10_	–	0.62	0.71	0.47	0.07	–	0.30	0.47	0.69	−0.03	–	0.63	0.66	0.67	−0.11
SO_2_		–	0.57	0.37	−0.26		–	0.69	0.31	0.09		–	0.69	0.60	0.00
NO_2_			–	0.42	0.01			–	0.51	−0.13			–	0.59	−0.33
CO				–	−0.34				–	−0.30				–	0.02

## Supplementary Materials

The following are available online at http://www.mdpi.com/1660-4601/15/6/1102/s1, Figure S1: Monthly ratios of PM2.5/PM10 in three cities during episode and non-episode days.
