# Supplementary material for: Particulate Matter and Gaseous Pollutions in Three Metropolises along the Chinese Yangtze River: Situation and Implications
Source: Int J Environ Res Public Health. 2018 May 28;15(6):1102. doi: 10.3390/ijerph15061102 (PMC6025567; doi:10.3390/ijerph15061102)
Supplement: Supplementary file 1 [file ijerph-15-01102-s001.pdf]

International Journal of *Environmental Research and Public Health*

Supplementary Materials: Particulate Matter and Gaseous Pollutions in Three Metropolises along the Chinese Yangtze River: Situation and Implications

Mao Mao, Xiaolin Zhang * and Yan Yin

Key Laboratory of Meteorological Disaster of Ministry of Education, Joint International Research Laboratory of Climate and Environment Change, Collaborative Innovation Center on Forecast and Evaluation of Meteorological Disasters, Key Laboratory for Aerosol-Cloud-Precipitation of China Meteorological Administration, Nanjing University of Information Science & Technology, Nanjing 210044, China; mmao@nuist.edu.cn (M.M.); yinyan@nuist.edu.cn (Y.Y.)

* Correspondence: xlnzhang@nuist.edu.cn; Tel.: +86-25-5869-9773

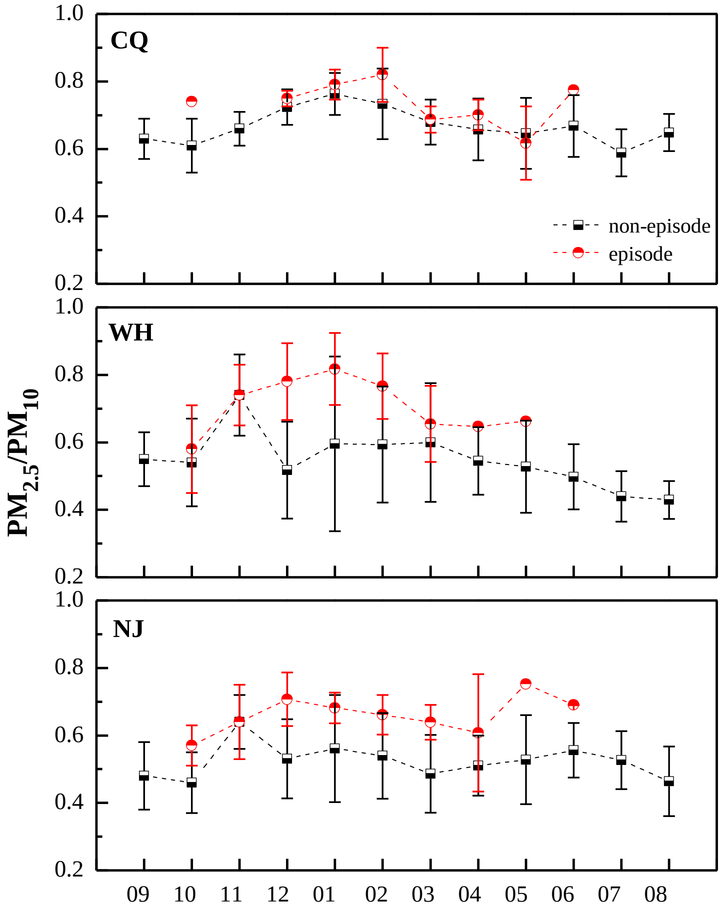

Figure S1. Monthly ratios of $PM_{2.5}/PM_{10}$ in three cities during episode and non-episode days.

© 2017 by the authors. Submitted for possible open access publication under the terms and conditions of the Creative Commons Attribution (CC BY) license (http://creativecommons.org/licenses/by/4.0/).
